# Menstrual hygiene management practice and its associated factors among in-school adolescent girls in Western Ethiopia

**DOI:** 10.1186/s40834-022-00196-7

**Published:** 2023-01-03

**Authors:** Naol Daniel, Gemechu Kejela, Firehiwot Fantahun, Markos Desalegn, Fantahun Guteta

**Affiliations:** Department of Public Health, Institute of Health Sciences, Wollega University, Nekemte, Ethiopia

**Keywords:** Menstruation, Hygiene, Adolescent girls, Secondary school, Gimbi town, Ethopia

## Abstract

**Background:**

Adolescent girls should practice good menstrual hygiene to enhance their health and educational attainment. However, socio-cultural restrictions and limited water, sanitation, and hygiene in school environments continued to make it difficult for in school adolescent females to practice good menstrual hygiene management. So, the main aim of this study was to assess menstrual hygiene management practice and its associated factors among in-school adolescent girls in the secondary schools of Gimbi town, western Ethiopia.

**Methods:**

A school-based cross-sectional study was conducted among 378 adolescent girls in Gimbi town secondary schools. The study participants were selected by using stratified random sampling techniques. Pretested self-administered Afan Oromo questionnaire with sociodemographic characteristics, information and knowledge about menstruation, and practice of menstrual hygiene management, as well as observational checklist to assess school environment were used to collect the data. Data were cleaned, coded, and entered into Epi-info version 7.2.0.4 and exported to SPSS version 25 for statistical analysis. Multivariable logistic regression analysis was used to identify factors associated with the practice of menstrual hygiene management and *P*-values less than 0.05 was used to declare statistical significance.

**Results:**

Out of 378 respondents, 163 (43.1%) (38–48) of adolescent girls had good menstrual hygiene management practice. Being urban resident (AOR = 3.48, (95% C.I:1.99–6.08), having mother with secondary level of education (AOR = 2.71, 95%CI: 1.15,6.42), having mother with educational level of college and above (AOR = 3.30, 95%CI1.28,8.50), having discussion about menstruation with parents (AOR = 2.42,95%CI:1.45,4.04), and having knowledge about menstruation (AOR = 2.94, 95% CI: 1.69–5.13) were factors associated with good menstrual hygiene management practice.

**Conclusion:**

In this study, good menstrual hygiene management practice is low among in school adolescent girls. Place of residence, maternal educational level, discussion about menstrual issue with parent, and having knowledge about menstruation were factors associated with good menstrual management practice. Stakeholders should give appropriate awareness and health education related to menstrual hygiene for adolescent girls at all levels.

## Background

Adolescence is a period of life when there are numerous opportunities for good health and when future patterns of adult health are formed. It is a time when they go through a serious of developmental tasks that mark their transition from childhood onwards, from ages ten to nineteen [1, 2]. Since changes that occur during this time can elicit excitement and concern, understanding what to expect at various stages of adolescence and early adulthood is crucial for healthy development [3]. Adolescent health is influenced by the interactions between prenatal, early childhood, unique biological, social-role changes, and sexual maturity [4].

From the many changes female adolescent experiences, menstruation is the natural event that signifies the maturation of the body [5, 6]. It is a monthly shedding of the layer of the uterine mucosa lining that happens once ovulation is not followed by fertilization, which occurs on average, every 28 days [6]. Menstrual Hygiene Management (MHM) is when women and adolescent girls use a clean material to collect menstrual blood, which can be changed in privacy as often as necessary for the length of menstruation, with soap and water for washing the body and reusable materials as needed and access to facilities for the disposal of used materials [7].

Even though menstruation is a natural event, female students may confront a variety of obstacles that prevent them from managing their period [8]. United Nations International Children’s Emergency Fund (UNICEF) report indicates that, every day 300 million women and girls do not have a comfortable environment, safe sanitary products, or a private area in which to manage their menstruation [9]. It was stigmatized, misunderstood, and girls felt uncomfortable and ashamed to discuss it in public, in class with their friends, and even at home with their families [10–12].

Globally, 2.3 billion people lack basic sanitation services, and in the least developed countries (LDC), only 27% of the population has access to water and soap for handwashing [13]. Managing periods at school is a significant challenge for adolescent girls who do not have access to these basic facilities [13, 14].

About half of the schools in low-income countries lack adequate drinking water, sanitation, and hygiene crucial for girls to manage their period [15]. In different countries of South Asia, 64, 43.1, and 41% of adolescent girls are absent from school due to menstruation [16–18] and nearly 70% of schoolgirls reported a lack of supplies for managing menstruation at school [19]. In Sub-Saharan Africa, 50 to 70% of girls miss an average of 1.6–2.1 days of school every month related to menstrual issues [20], and more than 50% of adolescent girls have inadequate menstrual hygiene practice, with a high proportion in rural areas [21]. In Ethiopia, the prevalence of safe menstrual hygiene management practice is 52.69% [22].

Despite efforts by various sectors, including international and local NGOs working in collaboration with the government, MHM is not integrated into existing efforts focusing on adolescent health [12], and it is not well implemented at all levels, particularly at the district and school levels [9]. Adolescent females in schools may experience a variety of challenges related to menstrual hygiene management because, it is not well understood and the factors associated with it are not well addressed by previous studies [8, 12, 20, 23]. In addition, even if studies are conducted on the topic in different parts of the country, to the search of the investigators, there is no study conducted on the same topic in the study area, so the prevalence of the problem is not known in the study area, and also, context specific/area related factors are not studied. Therefore, this study was intended to identify menstrual hygiene management practice and associated factors among in-school adolescent girls in Gimbi town.

## Methods and materials

### Study design, setting and population

A school-based cross-sectional study was conducted among 383 secondary school adolescent girls of Gimbi town from May 15/2022 to June 1/2022. Gimbi is the capital town of west Wollega zone located at 431 km to the west of Addis Ababa. The total population of the town is estimated to be 30,918 of which 15,265 are females [24]. Data from the Gimbi town education office show that there are six high schools (4 governmental and 2 private) in the town. The total number of female students enrolled in grade 9th and 10th for the academic year 2021/2022 were 2043. The number of girls studying in the 9th and 10th grades were 1071 and 972 respectively [25]. All regular school adolescent girl of grades 9th and 10th from all public and private schools in Gimbi town who met the inclusion criteria were the study population for this study. Students who had hearing and sight problems, had mental disorders, those who were absent during data collection, and who were seriously ill were excluded from the study.

### Sample size determination and sampling procedure

The sample size was calculated using a single population proportion formula considering the following assumptions; 34.7% prevalence of good practice of MHM from the previous study conducted in Holeta town, Ethiopia [26], 95% confidence level, 5% margin of error, and 10% non-response rate. By using the formula, $\textrm{n}=\frac{{\left(\textrm{Z}\alpha /2\right)}^2\textrm{p}\left(1-\textrm{p}\right)}{{\textrm{d}}^2}$, the final sample size becomes 383.

Sample size was also calculated for factors associated with menstrual management practice, by using double population proportion formula as indicated in Table 1. However, since the sample size for the first specific objective (prevalence of MHM) is higher than the sample size calculated for the second specific objective (associated factors), the largest sample size was taken as the overall sample size for the study.

**Table 1:** Sample size calculation for the second specific objectives (associated factors) by using STAT CALC of EPI INFO version 7.2.4.0

S.No.	Associated factors/variables	% of outcome in the un exposed group	% of outcome in the exposed group	The ratio of unexposed to exposed	AOR	Power %	CI%	Calculated sample size (n)	Ref
1	Female toilets kept locked inside	19.64	40.8	0.71	2.82	80	95	**171**	[26]
2	learn about menstrual hygiene at school	35.8	57.9	1.037	2.47	80	95	**175**	[27]
3	Residence	22.2	42.8	0.737	2.62	80	95	**186**	[26]

Stratified sampling technique was used to select 383 study participants. Initially, all high schools found in the town were stratified as private and public. As a result, there were 4 publics and 2 private high schools. Then, number of female students in both private and public schools were identified and proportionally allocated to the sample size. Finally, study participants were selected by simple random sampling from each section by using female students list taken from respective schools.

### Data collection tools and procedure

After reviewing relevant literatures, a self-administered structured questionnaire which had three parts: socio-demographic, information and knowledge about menstruation, and MHM practice was developed. It was prepared in English and then translated into Afan Oromo and retranslated to English by different proficient speakers of both languages, to maintain its consistency. Observational checklist which was developed by UNICEF and contextualized to the local setting [28] was also used to assess school environment. Four experienced female Nurses and two supervisors (Health officers) were engaged in data collection and supervision respectively for 2 weeks. Regarding the observational checklist, three observers in collaboration with cleaners, girls’ club leaders and unit leaders from each school were observed the schools.

### Data quality control

To ensure the quality of data, training was given for data collectors and supervisors with Nurse Professionals (BSc). A pre-test was conducted 1 week before data collection on 5% of the sample size in Darge high school, Nekemte town, which has the same socio-demographic characteristics as the study population. Based on the result of the pretest, corrections and modifications were made to the questionnaire. The supervisors and data collectors were given orientation on the objective of the study before the questionnaires were filled by respondents. Supervisors checked the errors, and completeness of the data, every day at the end of data collection.

### Measurements

To measure the level of knowledge about menstruation, the item responses were recoded as 1 for correct answer and 0 for wrong answer or don’t know, then the sum score of knowledge was calculated out of 12 knowledge questions. The mean value was calculated and the mean score (7.9 ± 1.96) designated as cut off point. Then respondent who scored below mean value were classified as having poor knowledge about menstruation, and those who scored above mean were considered to have good knowledge about mensuration.

Similarly, the level of menstrual hygiene management practice was calculated by first recoding the responses into 1 (right answers) and 0 (wrong or don’t know), and then summing the score of practice out of 12 practice questions, the mean score (6.2 ± 1.77) was used as the cutoff point. The respondents who scored above mean value were labeled as having good practice, while those who scored below mean value were labeled as having poor practice.

### Data processing and analysis

Data were coded, cleaned, and processed to identify missing values, outliers and inconsistencies. The coded data were checked for completeness and entered into EPI INFO version 7.2.4.0 and exported to SPSS version 25 for statistical analysis. Descriptive statistics were presented using frequency and percentages.

Logistic regression was also conducted to identify associated factors with MHM practice. First, bivariable logistic regression analysis was conducted to identify candidate variables for multivariable logistic regression. Accordingly, variables with *p*-value less than 0.25 at bivariable logistic regression were considered as candidate variables for the final model (Multivariable logistic regression).

In multivariable logistic regression, residence, mother’s educational level, having discussion about menstrual hygiene issues with parents, and having knowledge about menstruation were significantly associated with MHM practice at *p* < 0.05. Odds ratio at 95% confidence interval was used to determine the strength of association. Hosmer-Lemeshow Goodness of Fit Test result showed *P* > 0.05, indicating that the model is a good fit. Presence of multicollinearity was detected by VIF, in which the result indicates VIF of less than ten, indicating that there was no severe multi collinearity among the independent variables.

## Results

### Socio-demographic characteristics

Out of 383 calculated sample size, a total of 378 high school female students participated from six high schools, with a response rate of 98.6%. The respondents’ mean age was (16.24 ± 1.01) with the minimum and maximum ages of 14 and 19 years old, respectively. The majority, 179 (47.4%) and 336 (88.9%) were protestant followers and Oromo ethnic group respectively. Most 348 (92.1%) of adolescent girls were single and 277 (73.3%) lives with both parents. Two-third 252 (66.7%) of the respondent were from urban and the respondent’s mother’s educational level and occupational status were primary education 105 (27.8%) and housewives 170 (45.0%) respectively. In terms of respondents’ fathers’ educational level and occupational status, 103 (27.2%) were secondary school and 95 (25.1%) were farmer respectively (Table 2).

**Table 2:** Socio-demographic characteristics of respondents (*n* = 378)

Variables	Categories	Frequency	Percentage
Age of respondent	14–16	245	64.8
	17–19	133	35.2
School type	Public	317	83.9
	Private	61	16.1
Grade level	9th	198	52.4
	10th	180	47.6
Residence	Urban	252	66.7
	Rural	126	33.3
Marital status	Single	348	92.1
	Married	17	4.5
	Others^a^	13	3.4
Living arrangements	Both parents	277	73.3
	Mother only	41	10.8
	Father only	13	3.4
	Relatives	29	7.7
	Others^b^	18	4.8
Mother educational status	Illiterate	75	19.9
	Read and write	70	18.5
	Primary school	95	25.1
	Secondary school	72	19.0
	College and above	66	17.5
Father educational status	Illiterate	44	11.6
	Read and write	55	14.6
	Primary school	75	19.8
	Secondary school	103	27.2
	College and above	101	26.7
Mother occupational status	Housewife	170	45.0
	Merchant	45	11.9
	Government employee	58	15.3
	Private employee	18	4.8
	Self-employed	35	9.3
	Daily laborer	52	13.8
Father occupational status	Farmer	95	25.1
	Merchant	36	9.5
	Government employee	92	24.3
	Private employee	27	7.1
	Self-employed	54	14.3
	Daily laborer	46	12.2
	Others^c^	28	7.4

Others: ^a^ Engaged, Divorced

Others: ^b^ Husband, Alone, Others: ^c^ No work, Religious leader, Driver

### Information and knowledge about menstruation

**Fig. 1:**
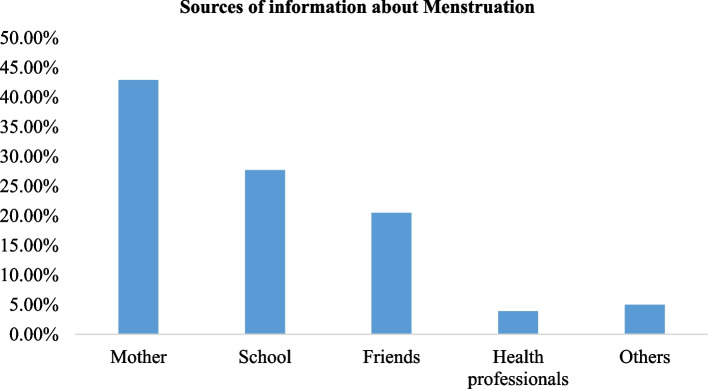
Sources of information about menstruation among in-school adolescent girls in Gimbi town, western Ethiopia, 2022 (*n* = 378)

The study found that 303 (80.2%) of the respondents heard about menstruation before attaining menarche, 130 (42.9%) from mother, 62 (20.5%) from friends, and 83 (27.4%) from school (Fig. 1). Out of 378 respondents, only 182 (48.1%) discussed about menstruation with their parents, out of this 106 (58.2%) and 58 (31.9%) discussed with their mothers and sisters respectively. For those who were not discussed the issue with their parents, the reason for not discussing were not habitual in 73 (37.2%) of respondents, and privacy or secrecy of the issues in 41 (20.9%) of the respondents (Table 3).

**Table 3:** Information and knowledge about menstruation of in-school adolescent girls in Gimbi town, western Ethiopia, 2022 (*n* = 378)

Characteristics of respondent	Categories	Frequency	Percentage
Age at menarche	< 12 Years	31	8.2
	13–15 Years	319	84.8
	16–19 Years	12	3.2
	Don’t Know	16	4.2
Heard about menstruation before menarche	Yes	303	80.2
	No	75	19.8
Discuss about menstrual hygiene issues with parents	Yes	182	48.1
	No	196	51.9
Discuss about menstrual hygiene issues with friends	Yes	333	88.1
	No	45	11.9
Know sanitary pad in the markets	Yes	352	93.1
	No	26	6.9
Cause of menstruation	Hormone	211	55.8
	Pathological	23	6.1
	Curse of God	48	12.7
	Don’t Know	96	25.4
Source of menstrual blood	Uterus	240	63.5
	vagina	26	6.9
	Bladder	21	5.6
	Abdomen	18	4.8
	Don’t know	73	19.3
Length of the normal menstrual bleeding	< 2 Day	66	17.5
	2–7 Days	219	57.9
	> 7 Days	41	10.8
	Don’t Know	52	13.8
Normal duration of the menstrual cycle	< 20 Days	78	20.6
	20–35 Days	217	57.4
	> 35 Days	19	5.0
	Don’t Know	64	16.9
Know that there is foul-smelling	Yes	255	67.5
	No	123	32.5
Know that menstrual blood is unhygienic	Yes	216	57.1
	No	162	42.9
Know that Pain during menstruation means that someone is not sick	Yes	261	69.0
	No	117	31.0
Know that Menstruation is not a lifelong process	Yes	362	95.8
	No	16	4.2
Learned about menstruation in school	Yes	232	61.4
	No	146	38.6
Overall knowledge about menstruation	Good	245	64.8
	Poor	133	35.2

### Menstrual hygiene management practice

**Fig. 2:**
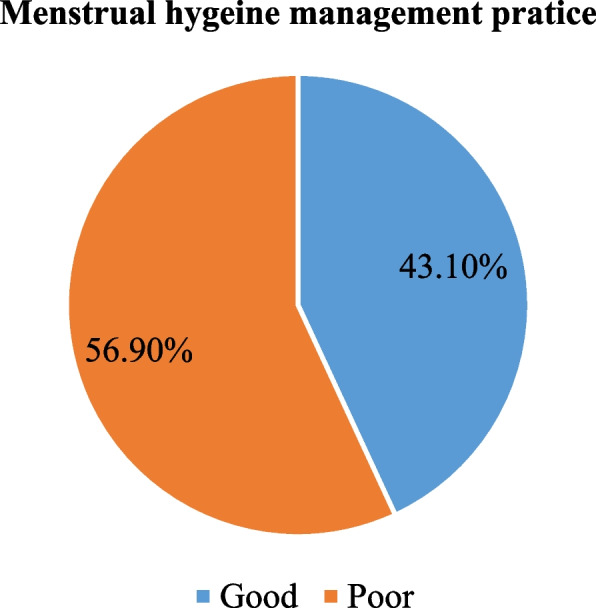
Menstrual hygiene management practice among in-school adolescent girls in Gimbi town, western Ethiopia, 2022 (*n* = 378)

Out of the total respondents, 163 (43.1%) (95% CI: 38–48) had good menstrual hygiene management practice. The majority, 365 (96.6%) of girls used absorbent materials during their last menstrual period and, 307 (84.1%) used disposable sanitary pads. In this study, 13 adolescent girls were not used sanitary materials during their last menstrual period. Of these 8 and 3 were due to the cost and unavailability of sanitary pads respectively. Out of 365 respondents, 349 (95.6%) of them changed their absorbent material during menstruation. More than half, 197 (56.4%) of the respondents changed their absorbent material three and more per day during the last menstrual period. Of 152 respondents who changed their absorbent material at school, only 23 (15.1%) of them did so every day during their last menstrual period. Out of 99 respondents who used reusable sanitary pads, 31 (31.1%) of them dry-washed reusable pads in the shade inside. The majority 251 (68.1%) disposed used sanitary pads in toilet. Of 365 girls who used sanitary pads during menstruation, 115 (31.5%) and 75 (20%) wrapped used pads to dispose them in plastic bags and paper respectively. One hundred sixty-two (44.4%) did not wrap used pads at all by any things (Fig. 2). Among those who used reusable sanitary pads, 31 (31%), 28 (28%), and 12 (12%) dried reusable sanitary pads in the shade inside, in the sunlight outside, and in the sunlight inside, respectively. In this study, 130 (35.6%) adolescent girls stored new and/or reusable absorbents alongside their regular clothes, 89 (24%) in drawers, and 40 (11%) in bathrooms (Table 4).

**Table 4:** Menstrual hygiene management practice among in-school adolescent girls in Gimbi town, western Ethiopia, 2022 (*n* = 378)

Characteristics of respondent	Categories	Frequency	Percentage (%)
Used sanitary materials (378)	Yes	365	96.6
	No	13	3.4
Sanitary material used (365)	Disposable sanitary pad	307	84.1
	Reusable sanitary pad	99	27.1
	Underwear only	8	2.2
	Disposable piece of cloth	26	7.1
	Use no sanitary pad	13	3.4
Frequency of changing absorbent (365)	Once	34	9.7
	Twice	118	33.8
	= > three times	197	56.4
Clean genitalia (378)	Yes	347	91.8
	No	31	8.2
Frequency of cleaning genitalia (347)	Once	71	20.5
	Twice	105	30.3
	Three times	99	28.5
	More than three times	72	20.7
Medium used for genital cleaning (347)	Only water	149	42.9
	Soap and water	198	57.1
Use reusable sanitary pad (365)	Yes	99	27.1
	No	266	72.9
Medium used to wash reusable clothes (99)	Only Water	5	5.2
	Soap and water	91	94.8
Take bath during menstrual period (378)	Yes	268	70.9
	No	110	29.1
Frequency of bathing (268)	<= two times in a day	164	61.2
	> Two times in a day	105	38.8
Change sanitary pad at school (365)	Yes	152	41.6
	No	213	58.4
Dispose of used menstrual materials (365)	In toilet /toilet	251	68.8
	Open field	21	5.8
	In waste bin	64	17.5
	Burned	17	4.7
	Others	12	3.3
Overall MHM Practice (378)	Good	163	43.1
	Poor	215	56.9

Others: buried, hidden Elsewhere

### School absenteeism and its reasons related to menstruation

Out of a total of 378 respondents, 125 (33.1%) did not attend school for an average of 1–2 days per month during menstruation. This was due to fear of leakage in 94 (75.2%) of respondents, can’t access sanitary pads in 22 (17.6%) of the respondents, no suitable place for pad exchange in 19 (15.2%) of respondents, and abdominal pain in 51 (40.8%) of the respondents (Table 5).

**Table 5:** School absenteeism and its reasons among in-school adolescent girls in Gimbi town, western Ethiopia, 2022 (*n* = 378)

Characteristics of respondent	Categories	Frequency	Percentage
Did not go to school during your menstruation (378)	Yes	125	33.1
	No	253	66.9
Average days absent from school monthly due to menstruation (125)	1–2 days	94	75.2
	2–3 days	22	17.6
	3–4 days	9	7.2
Reason for absence (125)	Lack of water in the school	12	9.6
	Lack of separate toilet	2	1.6
	Can’t access sanitary pad	19	15.2
	No suitable place	19	15.2
	Fear of leakage	22	17.6
	Abdominal pain	51	40.8

### Water, sanitation, and hygiene observation

#### Water observations

Piped water in the school was the primary source of water for all of the schools. During the observation, four out of six schools’ main water sources were not functional. Every school had water taps that were easily accessible to the youngest students, but not to children with physical disabilities. Three out of six high schools lack a drinking water storage container, and of those that have, only one is functioning.

#### Sanitation observations

On their compounds, all high schools have gender-specific toilets. There was a pit toilet with the slab in each of the six schools. The majority of the toilets were in use and partially functional, while others were not functional. The majority of the toilets had evident signs of feces and urine on the ground. There was a bad odor and most of them are classified as somewhat clean and not clean. In Some toilets, the day light inside was the same as outside because the toilet had a broken door. And most of the toilets were somewhat dark that is less visibility on the inside but possible for girls to see if their uniforms were stained. Some of the toilets had doors that could be locked from the inside, while others had no doors at all. In most toilets, there was no container for disposing used sanitary pad. There were no accessible toilets for those with disabilities. Only one private school had private room for girls to bath or replace sanitary pads, but it was not functional.

In fewer visited school facilities, there was a waste pit that was too full and there was no incinerator/pit to burn used sanitary pad and other dry wastes. There was no school with a drainage system for removing waste water.

#### Hygiene observations

All high schools have running water from a piped system and hand-washing facilities. The hand-washing facilities were located a great distance away from the toilet blocks. However, at the time of the visit, only few of the facilities seen had water accessible for hand washing at the handwashing facilities. During the observation time, no water, soap, or ash were available in any of the six schools. Some of the handwashing stations were accessible to younger children but not disabled children.

### Factors associated with menstrual hygiene management practice

In binary logistic regression analysis, school type, residence, marital status, living arrangement, mother educational level, father educational level, mother occupational status, father occupational status, heard about menstruation before attaining menarche, discussing about menstruation with parents, discussing about menstruation with friends and overall knowledge about menstruation were variables with *p*-value < 0.25, and considered as candidate variables for the multivariable logistic regression analysis.

In multivariable logistic regression, after controlling the confounding effects, it was found that adolescent girls who were from urban were three and half times (AOR = 3.48, 95%CI: 1.99,6.08) more likely to have good practice of menstrual hygiene management compared to rural residents. This study also found that adolescent girls who had mothers with secondary educational level were 2.7 times (AOR = 2.71, 95% CI: 1.15, 6.42), and those who had mothers with college and above educational level were 3.3 times (AOR = 3.30, 95% CI: 1.28, 8.50) more likely to have good practice of menstrual hygiene management compared to those whose mothers cannot read and write. The result also indicates that, adolescent school girls who discussed about menstruation with their parents were 2.4 times (AOR = 2.42, CI: 1.45, 4.04) more likely to practice good MHM compared to their counterparts. Furthermore, this study also revealed that adolescent girls who had good knowledge about menstruation were 2.9 times (AOR = 2.94, 95% CI: 1.69, 5.13) more likely to practice good MHM compared to those who had poor knowledge (Table 6).

**Table 6:** Multivariable logistic regression analysis for factors associated with menstrual hygienic practice in-school adolescent girls in Gimbi town, western Ethiopia, 2022

Variables	Category	MHM Practice		COR (95% CI)	AOR (95% CI)
		Good	Poor		
School type	Private	31	30	1	1
	Public	132	185	0.69 (0.40–1.20)	0.70 (0.35–1.41)
Residence	Rural	30	96	1	1
	Urban	133	119	3.58 (2.22–5.77)	3.48 (1.99–6.08)
Marital status	Single	147	201	1	1
	Married	10	7	1.95 (0.73–5.25)	1.90 (0.56–6.43)
	Others	6	7	1.17 (0.39–3.56)	1.05 (0.24–4.62)
Mother education	Illiterate	19	56	1	1
	Read and write	21	49	1.26 (0.61–2.62)	1.17 (0.48–2.85)
	Primary school	44	51	2.45 (1.32–4.91)	2.24 (0.99–5.09)
	Secondary school	39	33	3.48 (1.74–6.99)	2.71 (1.15–6.42)
	College and above	40	26	4.53 (2.21–9.29)	3.30 (1.28–8.50)
Father education	Illiterate	12	32	1	1
	Read and write	17	38	1.19 (0.50–2.86)	1.40 (0.48–3.93)
	Primary school	27	48	1.50 (0.66–3.40)	1.80 (0.67–4.80)
	Secondary school	50	53	2.52 (1.17–5.42)	1.70 (0.67–4.21)
	College and above	57	44	3.46 (1.60–7.47)	2.37 (0.92–6.08)
Mother occupation	Daily laborer	19	33	1	1
	House wife	67	103	1.13 (0.59–2.15)	1.20 (0.55–2.64)
	Merchant	21	24	1.52 (0.65–3.43)	1.55 (0.58–4.30)
	Government employee	31	27	1.99 (0.93–4.28)	1.15 (0.41–3.19)
	Private employee	10	8	2.17 (0.73–6.44)	1.22 (0.31–4.77)
	Self employed	15	20	1.30 (0.54–3.13)	0.87 (0.30–2.51)
Father occupation	Daily laborer	18	28	1	1
	Farmer	36	59	0.95 (0.46–1.96)	0.94 (0.39–2.23)
	Merchant	19	17	1.74 (0.72–4.20)	1.56 (0.56–4.41)
	Government employee	48	44	1.70 (0.83–3.49)	1.52 (0.64–3.64)
	Private employee	14	13	1.68 (0.64–4.37)	1.22 (0.36–4.10)
	Self employed	20	34	0.92 (0.41–2.06)	1.20 (0.46–3.18)
	Others	8	20	0.62 (0.23–1.71)	0.49 (0.15–1.64)
Heard about MHM before menarche	Yes	142	161	2.27 (1.31–3.94)	1.68 (0.87–3.24)
	No	21	54	1	1
Discuss MHM issue with parents	Yes	102	80	2.82 (1.85–4.30)	2.42 (1.45–4.04)
	No	61	135	1	1
Discuss MHM issue with friends	Yes	148	185	1.60 (0.83–3.09)	1.21 (0.54–2.76)
	No	15	30	1	1
Know Sanitary pads in market	Yes	155	197	1.77(0.75–4.18)	2.11 (0.73–6.08)
	No	8	18	1	1
Learned menstruation in school	Yes	106	126	1.31 (0.86–2.00)	1.52 (0.89–2.59)
	No	57	89	1	1
Knowledge about menstruation	Good	131	114	3.63 (2.27–5.80)	2.94 (1.69–5.13)
	Poor	32	101	1	1

*AOR* Adjusted odds ratio, *CI* Confidence interval; 1: Reference category, *p* < 0.005

## Discussion

This study aimed to assess the level of menstrual hygiene management practice and to identify significant factors associated with practice of menstrual hygiene management in-school adolescent girls of Gimbi town. Out of 378 respondents, 163 (43.1%) (95%CI: 38–48) adolescent girls had good menstrual hygiene management practice. Residence, mother’s educational status, discussion about menstrual hygiene issues, and knowledge about menstruation were significant variables associated with the good practice of menstrual hygiene management.

According to this study, the practice of MHM was 163 (43.1%) (95% CI: 38–48). This is comparable with findings in Harari region (44.2%), Ambo (46.7%), and southern Ethiopia (39.7%) [29–31]. However, it is lower when compared to studies in Hararge (58.3%), North-eastern Ethiopia, (52.9%), and Nepal (67%) [32–34]. The finding of the current study is higher than research done in Holeta (34.7%), and Bahir dar (24.5%) [26, 35]. The disparity may be due to differences in the research area, study population, and measurement. This is supported by the observational finding that, the schools had poor WASH facilities.

This study found that adolescent girls from urban areas were 3.4 times more likely to practice good menstrual hygiene management than those from rural areas. This is consistent with studies undertaken in Harari and East Hararge [31, 33]. This may be due to urban adolescents are near to information and services related to sexual and reproductive health issues, including menstrual issues compared to those who came from rural area.

Mother’s educational level was significantly associated with menstrual hygiene management practice. Girls whose mothers had a secondary educational level were 2.7 times more likely to practice good menstrual hygiene management than respondents with illiterate mothers. This is consistent with the studies conducted in Sebeta town, and Bahirdar [35, 36]. Furthermore, when compared to respondents with illiterate mothers, adolescent girls whose mothers had college and above educational level were 3.3 times more likely to practice good menstrual hygiene management. This finding is consistent with the studies conducted in North India and Addis Ababa [27, 37]. This might be due to the fact that literate mothers can give an information about menstrual hygiene management practice for their daughters, and also, mother-daughter communication about menstruation is higher among literate mothers compared to those who are illiterate.

It was found that, adolescents girls who discussed about menstruation with their parents were 2.4 times more likely to practice good MHM than their counterparts. This is consistent with the studies conducted in Addis Ababa, Dessie, Holeta, Ambo, and systematic review and meta-analysis conducted in Ethiopia [26, 27, 29, 32, 38]. This could be because adolescent females who discuss menstruation with their parents may gain knowledge about menstruation that in turn increase the practice of good MHM. This also helps adolescent girls to get experience and money to buy a sanitary pad.

When compared to their counterparts, those with knowledge of menstruation were 2.9 times more likely to practice good menstrual hygiene management. This is in line with the research undertaken in Nigeria, Mehalmeda, and North Wollo Zones [39–41]. This is important because without enabling factors that might disturb the association, there is a normal expectation and positive relationship between knowledge and practice.

This study used a cross-sectional design, so that, identifying causal relationships is difficult. The sensitivity of menstrual issues may lead to social desirability bias. Furthermore, employing close-ended questions during data collection may limit further options and more sophisticated judgment.

## Conclusions and recommendations

The study found that 43.1% of adolescent girls had good menstrual hygiene management practice. Being from urban, having mothers with higher educational level, discussing menstruation with parents, and having knowledge about menstruation were associated with good menstrual hygiene management practice. There was also lack of adequate WASH facilities in school compounds.

This indicates that educating mothers, enhancing menstrual hygiene knowledge, and encouraging discussion of menstrual hygiene management issues, particularly in rural areas, should be priority intervention areas to improve menstrual hygiene management practice. As a result, to address these issues, health care facilities, schools, and other stakeholders should design appropriate awareness raising and health education programs for adolescents on menstrual hygiene at all levels. They should also create a supportive environment that encourages students to handle their menstrual hygiene safely.

## Data Availability

Data will be available upon request from the corresponding author.

## Abbreviations

AOR: Attribute Odds Ratio
BSc: Bachelor of Science
CI: Confidence Interval
COR: Crude Odds Ratio
CTAG: Global Terminology Action Group
MHM: Menstrual Hygiene Management
NGO: Non-Governmental Organization
LDCs: Least Developed Countries
ORHB: Oromia Regional Health Bureau
OREB: Oromia Regional Education Bureau
PI: Principal Investigator
RTIs: Reproductive Tract Infections
SPSS: Statistical Package for Social Sciences
SRS: Simple Random Sampling
UNICEF: United Nations International Children’s Emergency Fund
VIF: Variance Inflation Factor
WASH: Water, Sanitation and Hygiene
WHO: World Health Organization
